# Effect of intensive glycaemic control on moderate hypoglycaemia and ICU length of stay in severe traumatic brain injury

**DOI:** 10.1186/s13054-018-2046-5

**Published:** 2018-05-23

**Authors:** Rafael A. Núñez-Patiño, Andres Zorrilla-Vaca, Daniel Agustin Godoy

**Affiliations:** 1 Faculty of Health Sciences, School of Medicine, Pontificia Universidad Javeriana, Cali, Valle del Cauca Colombia; 2 Faculty of Health, Universidad del Valle, Hospital Universitario del Valle, Cali, Colombia; 3 Neurointensive Care Unit Sanatorio Pasteur, Intensive Care Unit, Hospital San Juan Bautista, San Fernando del Valle de Catamarca, Catamarca Argentina

Glycaemic alterations are prevalent and modifiable secondary insults with detrimental consequences in neurocritically ill patients. Without discerning whether hyperglycaemia is a marker of lesional severity or the cause of brain damage, its association with poor results is clear due to its deleterious effects by promoting inflammation, thrombosis of the microcirculation and immunosuppression, among others. On the other hand, both the duration and depth of an episode of hypoglycaemia have a negative influence on final outcome. Despite this, a general consensus regarding the best glycaemic control in traumatic brain injury (TBI) has not been established yet.

We read with great interest the article “Glycaemic control targets after traumatic brain injury: a systematic review and meta-analysis” by Hermanides et al. [1]. Although the authors attempted to summarize the best evidence possible, we want to discuss some issues that may render their conclusions controversial. First, the authors did not perform sensitivity analysis to evaluate the effect of specific studies on the results, undoubtedly leading to wrong interpretations, thus limiting the validity of their study.

Second, after analyzing the data from the included studies and performing a sensitivity analysis using the leave-one-out method in Stata v.13, we found that a single study (Bilotta et al. [2]) was influencing the pooled effect size for the risk of moderate hypoglycaemia (blood glucose (BG) < 80 mg/dL) (Table 1), which the authors reported as non-significant in the first place (relative risk (RR) = 0.26; 95% confidence interval (CI) [0.00, 27.84], *P* = 0.57). By removing the study by Bilotta et al., the risk of moderate hypoglycaemia is higher with intensive glycaemic control (RR = 0.15; 95% CI [0.10, 0.23], *P* < 0.01). This is relevant because intensive glycaemic control has been associated with moderate and severe hypoglycaemia, which have been associated with poor outcomes.

**Table 1:** Sensitivity analysis comparing the risk of moderate hypoglycaemia (BG < 80 mg/dL) with intensive versus conventional glycaemic control

Study omitted	Year	RR	95% CI	*P* value	Heterogeneity (*I*^*2*^)	*P* for heterogeneity
Bilotta et al. [2]	2008	0.15	0.10, 0.23	< 0.01	11%	0.34
Coester et al. [3]	2010	0.27	0.00, 96.34	0.67	100%	< 0.01
Green et al. [4]	2010	0.27	0.00, 66.88	0.64	100%	< 0.01
NICE-Sugar [5]	2015	0.33	0.01, 12.31	0.54	99%	< 0.01
Van den Berghe [6]	2005	0.27	0.00, 50.32	0.63	100%	< 0.01
Combined [2–6]		0.26	0.00, 27.84	0.57	100%	< 0.01

*BG* blood glucose, *CI* confidence interval, *RR* relative risk

**Fig. 1:**
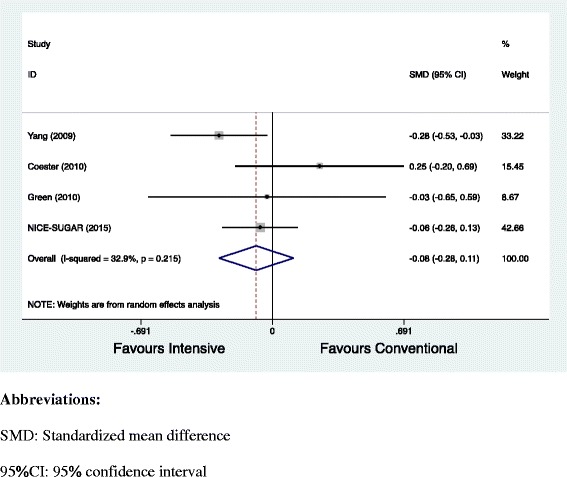
Forest plot comparing ICU length of stay in days between intensive versus conventional glycaemic control

Third, the included studies [2–5] consistently reported the ICU length of stay as a clinical outcome. The pooled effect size for this outcome was not estimated in the study. We found that there was no significant difference between intensive versus conventional glycaemic control with regard to ICU length of stay (standardized mean difference = − 0.08; 95% CI [− 0.28, 0.11], *P* = 0.39) (Fig. 1).

Finally, we have two simple questions. Microdialysis studies have revealed that lowering levels of glycaemia below 110 mg/dL is associated with the development of metabolic crises in a brain vulnerable to glucose deficit; therefore, should the definitions of hypoglycaemia not be reconsidered in this context? And is the dichotomized Glasgow Outcome Scale a good form to evaluate the final outcome in severe TBI?
