# Supplementary material for: Effect of intensive glycaemic control on moderate hypoglycaemia and ICU length of stay in severe traumatic brain injury
Source: Crit Care. 2018 May 23;22:134. doi: 10.1186/s13054-018-2046-5 (PMC5966873; doi:10.1186/s13054-018-2046-5)
Supplement: Related texts

# Reply: Authors’ response

**Authors:** J. Hermanides, M. P. Plummer, M. Finnis, A. M. Deane, J. P. Coles, D. K. Menon

We thank Rafael Núñez-Patiño and coworkers for their interest and comments on our article [1], to which we would like to respond. The authors have suggested the results lack validity as we did not perform a sensitivity analysis to evaluate the effect of specific studies. They have supported this assertion by performing a leave-one-out analysis—identifying one outlier study for the secondary outcome ‘moderate hypoglycaemia’—which results in a statistically significant association between moderate hypoglycaemia and intensive insulin therapy, in keeping with the association reported for our primary outcome, severe hypoglycaemia.

Given their analysis, we assume they have also confirmed that there are no outlier studies for the primary outcomes, i.e., mortality and severe hypoglycaemia. The presence of marked heterogeneity for the ‘moderate hypoglycaemia’ studies was clearly recognized and reported in our paper (I^2^ > 95%), with the influence of the Bilotta study clearly shown in the forest plot (Fig. 6 in [1]). We chose to analyse the data with a random effects model as it was clear from looking at the heterogeneity within patient populations, glycaemic control protocols and metrics of glycaemic control that we would not be estimating a common treatment effect, but rather the range of effects present in relevant studies. In keeping with Higgins, we do not believe heterogeneity allows removal of an offending study. To quote him, this approach “raises important questions about the validity of the subsequent meta-analysis, since removal of studies is tantamount to manipulation of the eligibility criteria” [6]. We believe reporting all relevant studies, along with heterogeneity, is a more valid approach, as this presents the reader with all relevant information, allowing the type of post-hoc subset analysis performed by Núñez-Patiño et al. Notwithstanding, these issues do not meaningfully alter the interpretation of our paper, as this post-hoc analysis of a secondary outcome parallels the findings of our primary outcome, i.e. both moderate and severe hypoglycaemia are associated with intensive insulin therapy [7].

Second, we thank Núñez-Patiño for performing a meta-analysis on length of stay for the different studies. We did not include this as an endpoint in our paper because ICU length of stay is not a patient-centred outcome and can be manipulated by many factors, including competing risk of death. The interpretation thus warrants caution.

Finally, we agree with Núñez-Patiño et al. that microdialysis glucose levels may be important, but we need to recognize that they do not address the risks of hyperglycaemia either cerebrally or extracranially. We have to be aware that ICU research is littered with interventions that made the numbers better but the patients worse.

To conclude, we agree that any meta-analysis should be interpreted with caution, especially in cases of marked heterogeneity. However, excluding studies from meta-analysis should be done based on predefined inclusion and exclusion criteria, and not because we do not ‘like’ the results.
